# Milk-Derived Extracellular Vesicles in Inter-Organism, Cross-Species Communication and Drug Delivery

**DOI:** 10.3390/proteomes8020011

**Published:** 2020-05-13

**Authors:** Rahul Sanwlani, Pamali Fonseka, Sai V. Chitti, Suresh Mathivanan

**Affiliations:** Department of Biochemistry and Genetics, La Trobe Institute for Molecular Science, La Trobe University, Bundoora, VIC 3083, Australia; 19168073@students.latrobe.edu.au (R.S.); P.Fonseka@latrobe.edu.au (P.F.); 19573709@students.latrobe.edu.au (S.V.C.)

**Keywords:** exosomes, extracellular vesicle proteome, animal extracellular vesicles, milk extracellular vesicles, cellular crosstalk, cross-species communication, interindividual communication, extracellular vesicle-based therapy, drug delivery

## Abstract

Milk is considered as more than a source of nutrition for infants and is a vector involved in the transfer of bioactive compounds and cells. Milk contains abundant quantities of extracellular vesicles (EVs) that may originate from multiple cellular sources. These nanosized vesicles have been well characterized and are known to carry a diverse cargo of proteins, nucleic acids, lipids and other biomolecules. Milk-derived EVs have been demonstrated to survive harsh and degrading conditions in gut, taken up by various cell types, cross biological barriers and reach peripheral tissues. The cargo carried by these dietary EVs has been suggested to have a role in cell growth, development, immune modulation and regulation. Hence, there is considerable interest in understanding the role of milk-derived EVs in mediating inter-organismal and cross-species communication. Furthermore, various attributes such as it being a natural source, as well as its abundance, scalability, economic viability and lack of unwarranted immunologic reactions, has generated significant interest in deploying milk-derived EVs for clinical applications such as drug delivery and disease therapy. In this review, the role of milk-derived EVs in inter-organismal, cross-species communication and in drug delivery is discussed.

## 1. Introduction

### 1.1. Extracellular Vesicles

**Figure 1:**
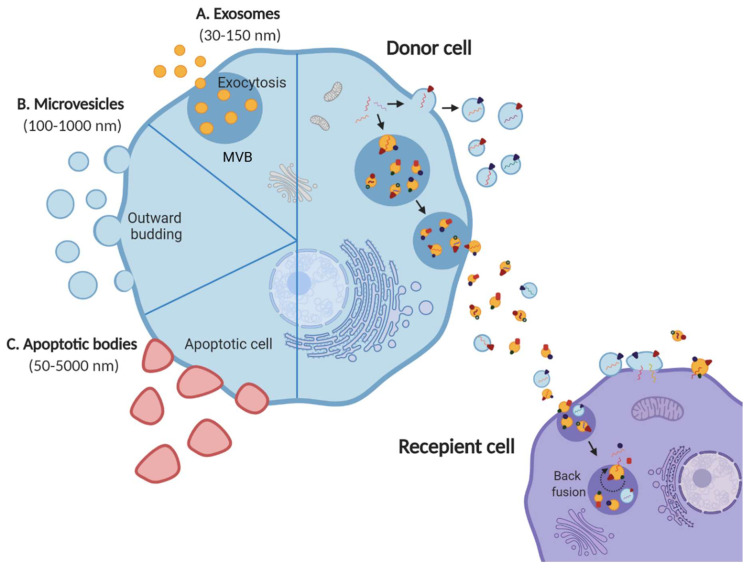
EV subtypes and role in mediating cellular crosstalk. Schematic representation of three main EV subtypes; (**A**) exosomes, (**B**) microvesicles, (**C**) apoptotic bodies released by all cell types into the extracellular space. Exosomes and microvesicles are released by live cells via exocytosis and outward budding, respectively. Apoptotic bodies, on the contrary, are released by apoptotic cells. EVs are known to mediate intercellular communication by carrying a diverse cargo of proteins, nucleic acids and lipids from the donor cell to the recipient. Adapted from [1].

Extracellular vesicles (EVs) are membrane vesicles secreted by all cell types into the extracellular space [1,2]. Although, it is known that apoptotic cells undergo blebbing and release apoptotic bodies, healthy cells indulging in active secretion of vesicles has been more recently discovered [3,4,5,6]. EVs shed by cells have been now classified into various categories: exosomes, apoptotic bodies and ectosomes or shedding microvesicles, depending on their biogenesis and origin [1,7,8]. Exosomes are vesicles ranging from 30–150 nm originating from the fusion of multivesicular bodies (MVBs) or late endosomes with plasma membrane, thus being endocytic in origin [9,10,11]. Apoptotic bodies (50–5000 nm) and ectosomes (100–1000 nm), contrarily, bud from the plasma membrane of apoptotic and live cells, respectively (Figure 1) [1,12,13]. Since their discovery, additional understanding of the molecular mechanisms regulating their biogenesis and secretion is emerging with their pivotal role in intercellular communication and pathophysiology [14]. Until two decades ago, intercellular communication was thought to be driven by direct cell–cell contact or secreted factors [4]. Over the years, EVs have emerged as more than mere garbage bags of cells to eliminate cellular debris and are understood to be involved in more sophisticated processes such as intercellular communication (Figure 1) [15,16,17,18]. Hence, EVs continue to garner more attention as their significance in health and disease unfolds.

### 1.2. EV Cargo

EVs carry a rich cargo comprised of proteins, nucleic acids, metabolites and lipids [1,19]. Even though, the presence of DNA in EVs secreted by all cell types is still controversial, packaging DNA in EVs-derived from cancer cells confers higher stability as compared to nascent secreted DNA [20,21,22,23]. EV cargo is often reflective of cell type of origin and can also vary depending on state of cell such as healthy, diseased, stressed and differentiation status [24,25,26,27].

EV proteome is the most well studied cargo content. They may contain proteins that are ubiquitous as well as specific to cell of origin [19,28]. EVs are highly abundant in endosome associated proteins involved in vesicle trafficking (Annexin, Rab, Flotilin and SNAREs). Some of these proteins such as TSG101 and Alix are involved in MVB biogenesis [4,10]. Heat shock proteins (HSPs) such as HSP70 and HSP90 are also known to be present in abundance and are involved in antigen binding and presentation to MHC proteins [29]. EVs are specifically found to be enriched with membrane proteins that are found in microdomains at the plasma membrane, particularly, tetraspanins (CD9, CD63, CD81 and CD82) and integrins [26,30,31]. Other membrane proteins such as EGFR, MHC I and MHC II may also be found in EV membranes [30]. Luminal cargo of EVs may also contain signal transducers, with a role in pathways such as Notch and Wnt signaling [32]. Furthermore, metabolic enzymes such as peroxidases, pyruvate and lipid kinases, cytochrome P450 and enolase-1 [10] and cytoskeletal proteins such as tubulin, actin and actin-binding proteins may also be present [19,33].

Though, the presence of proteins in EVs has been known since its discovery, the identifications of RNA including microRNA (miRNA) and mRNA created significant interest [34]. Transcriptome profiling of EVs has revealed presence of functional mRNA and miRNA in both free as well as bound states [35]. Functional transfer of these nucleic acids to recipient cells has shown to induce regulatory modulations and simulation of signaling pathways [34]. EVs are comprised of a lipid bilayer, however, the lipid content of EV cargo has been shown to have a greater role than just imparting structural integrity [36]. Lipids and lipid-related enzymes such as phospholipids, ganglioside GM3, sphingomyelin, ceramide, cholesterol and prostaglandins are found in EVs [37,38,39]. Lipid composition of EVs has been observed to be different than that of originating cell’s plasma membrane [37,39]. Like proteins and RNA, lipids in EVs too have been observed to have functional effect and mediate intercellular communication by simulating recipient cell’s signaling pathways. For instance, sphingomyelin in tumor-derived EVs has been shown to regulate angiogenic activity [40]. Taken together, EVs harbor a highly diverse cargo of biomolecules with potential role in mediating phenotypic alterations in recipient cells and tissues.

### 1.3. EVs in Biofluids

Biofluids such as urine [41], breast milk [42], amniotic fluid [43], cerebrospinal fluid (CSF) [44], saliva [45] and blood [46] consist of a wide variety of EVs secreted by different cell types. These EVs have been implicated in a plethora of uncharacterized functions, mainly depending on the identity of donor and recipient cells. Over the years, efforts to understand the function of EVs have highlighted their role in various physiological and pathological conditions. These EVs have been observed to be associated to a broad range of functions including signaling [47,48], protein clearance [49,50], establishing infections [51,52], immune modulation [53,54] and cancer [18,55]. The role of EVs in pathological processes and their presence in biofluids makes them a suitable target to be exploited as disease biomarkers in liquid biopsies, with a greater ease of access [24]. In this regard, the potential of using EVs enriched in CSF of patients with neurodegenerative disorders as disease biomarkers and for novel therapeutic alternatives has been demonstrated [44]. EVs in biofluids also have potential role in disease progression and pathology [56]. For instance, the role of EVs secreted by brain cancer cells in promoting metastasis by permeating the brain blood vessels and destructing the blood brain barrier has been demonstrated [57]. Furthermore, many studies have suggested impaired EV biogenesis and secretion as a consequence of a pathological condition. For instance, reduced EV secretion has particularly been observed in various neurodegenerative disorders such as Alzheimer’s and Huntington’s disease [58,59]. Contrastingly, enhanced EV secretion in tumor cells exposed to hypoxia is observed, primarily as a means to dispose metabolic waste and increase survival [60]. Hence, aggressive and metastatic tumor cells have been reported to exhibit enhanced EV release contributing to cancer-associated cachexia and pre-metastatic niche formation [61,62,63,64]. Similarly, studies have suggested that cancer therapeutics may enhance the secretion of EVs by tumor cells, which have a distinct cargo capable of inducing chemoresistance in recipient cells [65]. Taken together, these observations suggest that based on the pathological state, EV numbers and the cargo within are altered in biofluids. Although, various observations indicate EVs in biofluids as mediators of cellular crosstalk, more efforts are now underway to better understand their potential role in health and disease.

## 2. Milk as a Biofluid in Interindividual and Cross-Species Communication

Milk serves as a functionally dynamic source of nutrition in infants across mammals and is the most widely consumed natural beverage [66]. Milk has evolutionarily developed as a neonatal food ensuring early development, growth and health in the short and long term [67,68]. Colostrum, the first breast fluid produced before breast milk, is a rich source of nutritional factors and bioactive compounds. Among these, colostrum consists of growth factors, immunological factors, epithelial cells, neutrophils and leukocytes [69,70,71]. In addition, the bioactive compounds present in milk include antimicrobials, cytokines, chemokines, amino acids, casein fragments, antibodies and lipids that can have a vast range of effects in the recipient [68,72,73,74]. Milk is also known to be a source of commensal and probiotic bacteria found in infant gut [75,76]. More recently, it was realized that other than immune cells, milk is also a vector to transmit the maternal stem cells to the infant, which contribute to its neural development and immunity [67,77,78].

Milk as a biofluid with a complex composition of bioactive compounds and cells, aids in inter-individual communication from as early as neonatal stage in life through breastfeeding [72,79,80]. As milk consumption from other sources such as goat, sheep, but predominantly bovine, is continued through adolescence into adulthood, the communication becomes cross-species [68,79]. Milk is particularly highly abundant source of miRNA, which are known for their role in post-transcriptional gene regulation. Furthermore, the lactation specific and immune related proteins and miRNA in milk may also exert epigenetic modifications, eventually resulting in gene expression changes in the recipient cells [68,81,82]. These regulatory components in milk may mediate inter-individual communication crucial to early development in neonatal stages [68,72,76,83]. Supporting the beneficial aspect of the milk mediated communication, there are reports suggesting anticarcinogenic, antimicrobial, immunomodulatory and metabolic implications of milk amino acids and proteins such as TGF-β, casein, lactoferrin and lactoglobulin [84,85,86,87,88]. However, milk consumption, which is not species-specific, although is known to have benefits, may be detrimental as well [79,83,89,90]. Hence, there are now concerns over the regulatory implications of continued milk consumption in humans. For instance, miR148a, which is the most highly expressed miRNA in milk has identical bovine and human sequence. This DNA methyltransferase (DNMT) regulating miRNA may thus be able to govern gene expression pattern in consuming adults [91].

The idea of cross-kingdom, species and interindividual transfer of bioactive compounds via diet, is a recent avenue [92,93]. Although there are many studies suggesting interindividual and cross-species transfer of dietary miRNA through milk or plant consumption, the concept remains highly debated and controversial [93,94,95,96,97,98]. It has been argued that for these bioactive compounds to exert physiological responses in the recipient, oral bioactivity and systemic bioavailability remain a challenge [99]. The harsh enzymatic activity and acidity in the intestinal tract make it unfeasible for the components to survive in the stringent conditions [100]. However, EVs present in milk and other dietary sources have shown to protect such cargo from the harsh degrading conditions [94]. Milk EVs, enriched in bioactive compounds, particularly miRNA, have been identified in human and bovine mature milk and colostrum. Hence, EVs are thought to play a critical role in cross-kingdom, species and inter-individual communication [42,101,102,103]. Although the avenue continues to be explored, the process of milk EVs transferring miRNA between individuals and cross-species has been referred to as ‘natural microRNA in vivo transfection system’ [79].

### 2.1. Milk EVs Mediate Intercellular Communication

**Figure 2:**
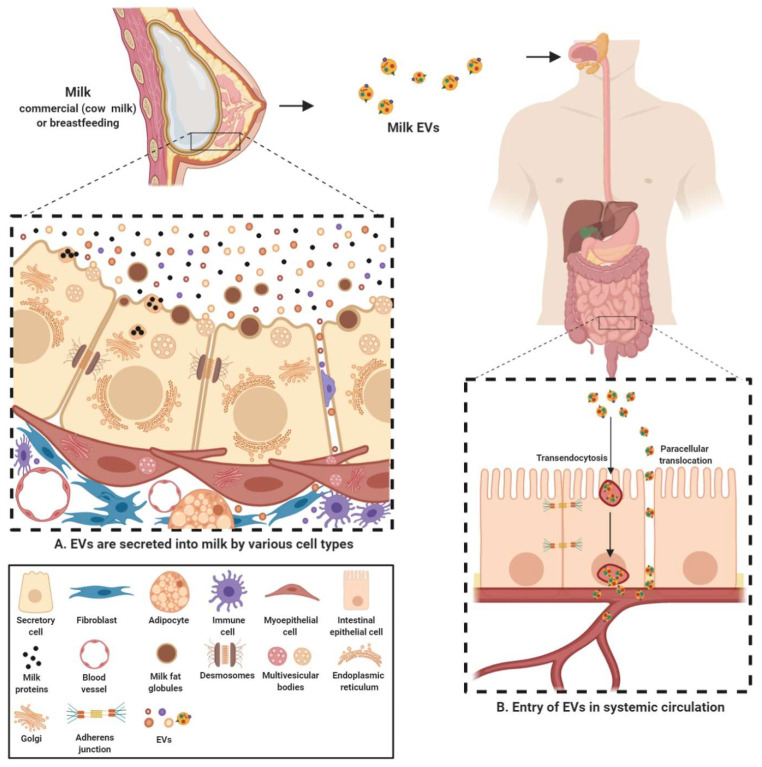
Milk EVs; source and role in cross-species inter-individual communication. (**A**). Milk EVs are highly heterogenous and are secreted into the milk by a variety of cells residing in the source organism’s mammary gland; mammary epithelial cells, immune cells, stem cells, bacteria and adipocytes. These vesicles are distinct from the MFGs and carry a diverse cargo of bioactive compounds. (**B**) Upon milk consumption, these EVs can survive the harsh, degrading conditions of recipient’s gut. These EVs are then either taken up by the IECs (transendocytosis) or pass through the leaky epithelial gut (paracellular translocation), eventually entering the systemic circulation.

Milk-derived EVs are nanosized signalosomes that have been identified in the colostrum and mature milk of humans and cows. EVs have also been found in milk of many other species including buffalos [104], pigs [105,106], and horses [107]. These EVs are present in vast abundance in the biofluid and carry a diverse cargo of proteins, lipids, RNA and other bioactive compounds [66,96,108,109,110]. They are known to be a fundamental means of intercellular communication between the breastfeeding mother and her newborn and now also cross-species due to consumption of milk in adulthood [111]. These lipid bilayer membrane vesicles are thought to be different form the milk fat globules (MFG) and originate from many different sources within the organism (Figure 2). EVs can originate from different cell populations present in the mammary gland-mammary epithelial cells, immune cells and mammary stem cells [83,108,112]. Furthermore, the source of these EVs can be bacterial, from the bacteria colonizing mammary glands or growing in milk [73,80].

Since the cargo contained in EVs is cell-type specific, milk EVs can contain a highly diverse cargo depending on multiple factors such as source organism, diet, health and well-being among many others [27,66,108,109]. Regardless of the source, the cargo transferred by milk EVs from source to recipient is indispensable in exerting a physiological effect. Milk EVs serve to provide a protective cover to the sequestered cargo and protect them from the harsh conditions in the gastro-intestinal tract. For instance, commercial milk-derived EVs have been demonstrated to be stable under conditions of low pH and boiling temperatures and freezing [81,113]. Furthermore, these harsh and degrading conditions of acidic and enzymatic activity had no significant effect on the levels of the otherwise labile cargo content [96]. Though it is unclear as why EVs in milk are more stable in harsh conditions, recent observations highlight that at least part of this effect can be attributed to calcium, in both raw and commercial milk, contributing to membrane integrity and stability (unpublished observations, Mathivanan lab). Other than milk EVs maintaining their integrity under challenging conditions in the gut, they may facilitate the delivery of the cargo contents into systemic circulation through intestinal epithelial cell (IEC) mediated transendocytosis [114]. In addition, milk EV uptake may also be facilitated by diffusion through the epithelial barrier; paracellular translocation, as has been observed recently in case of bacteria and EVs [115,116] (Figure 2). However, in vivo evidence supporting either of these mechanisms is currently lacking. It is worthy to note that, over the last few years, many studies have focused on the uptake of human and bovine milk EVs in various cell types. These studies have not only successfully demonstrated the uptake of milk EVs by IECs [105,114,117,118] but also macrophages [102] and vascular endothelial cells [119]. Milk EVs have also been demonstrated to be able to cross blood brain barrier. This ability of milk EVs make them bioavailable to reach distant sites in the body and hence has the potential to regulate gene expression in various tissues [103]. Hence, these inherent attributes make milk EVs a preferred vector for transmission of biomolecules and regulatory factors into systemic circulation.

### 2.2. Milk EV Cargo

**Figure 3:**
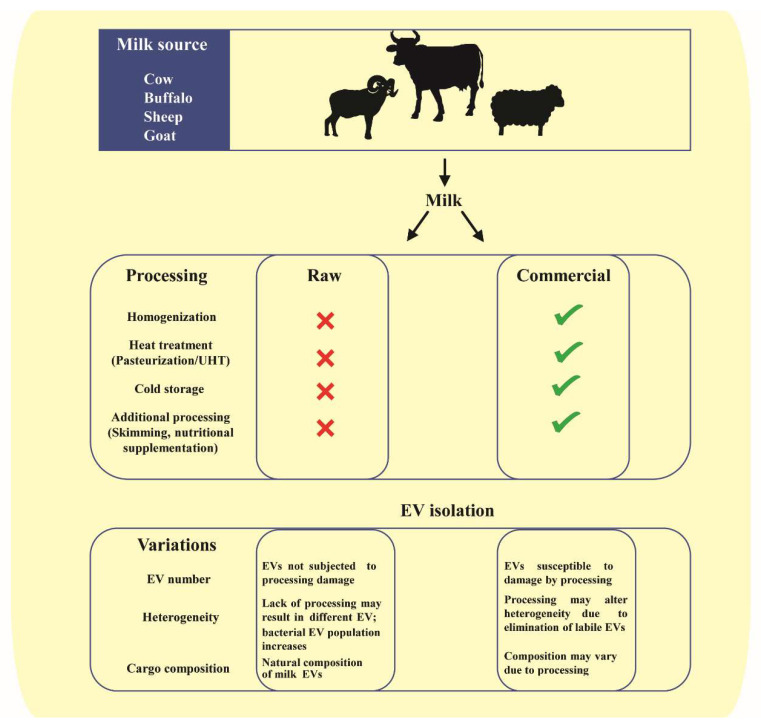
Schematic representation of differences in milk-derived EVs between raw and commercial milk. Raw milk from various species undergoes numerous processing steps before commercialization. These processes; homogenization, heat treatment, cold storage, skimming and nutritional supplementation lead to variations in milk-derived EVs. The isolated EVs from raw and commercial milk may thus differ in abundance, composition and cargo.

EVs in milk are derived from heterogenous cells, as described earlier, and harbor a diverse cargo. The EV and the cargo within can be altered based on the source of species, breed, diet and health state [27,66,104,106,108]. In the context of commercial and raw milk, the processing involving homogenization and pasteurization may also influence the EVs and their cargo (Figure 3). For instance, milk miRNA has been shown to be susceptible to processing and handling. Processes such as pasteurization and homogenization led to significant reduction in levels of milk miR200c and miR29b [120]. Furthermore, microwave has also been shown to reduce the miRNA and mRNA content of milk EVs, possibly due to EV damage and exposure to degrading enzymes [121]. While pasteurization might not significantly alter milk EV cargo, ultra-heat-treated milk EV cargo is significantly different than that of pasteurized and raw milk. Prolonged heat treatment at high temperature has been attributed to loss of EV cargo biological activity in some cases [103,122]. In corroboration with these observations, sonication too has been observed to result in a reduced miRNA cargo in milk EVs, possibly due to wreckage of vesicle membrane [82]. Furthermore, cold storage conditions have also been shown to significantly deplete the milk EV cargo [104]. Contradicting these observations, there are also studies indicating no remarkable change in levels of certain milk EV miRNA cargo in response to harsh conditions such as prolonged boiling and low pH when compared to untreated raw milk [96,104]. This might be attributed to the varying stability of EVs from raw and commercial milk sources under such degrading conditions of prolonged heating and acidity. Consistent with most of the literature, proteomic and transcriptomics analysis revealed significant differences in protein and RNA profiles in raw and commercial milk samples, respectively (Unpublished observations, Mathivanan laboratory). Even though observations from these early studies on effects of processing on EV cargo is compelling, further studies are required to define the precise effects of these processes on milk EVs and their cargo in terms of altered bioavailability and bioactivity. While, to date, several studies have probed the cargo of milk EVs, the functional role of the precise cargo is yet to be fully understood [66,103,111,123].

In addition to milk processing, the EV heterogeneity and composition can also vary based on the isolation method employed. Though differential centrifugation coupled with ultracentrifugation remains the most widely used method for harvesting milk-derived EVs [42,66,123], various other methods such as precipitation [118,121] and size exclusion chromatography [124] are also employed. However, these isolation methods can result in different EV number and composition from the same milk samples [125]. Hence, additional studies are needed to compare the isolation methods to harvest milk EVs and study the variation in cargo and functional role.

Proteins present in the milk EVs have been broadly categorized as membrane or luminal. The initial study revealed that milk EV proteome is much more diverse than that of MFG membrane. Milk EVs and MFGs have been shown to have distinct secretion pathway. Further, while the MFG membrane has been shown to be mostly enriched with proteins implicated in the lipid secretion pathways, milk EV proteome consisted of proteins, which reflect other functions of secretory cells [108,123]. Consistent with mammalian cell-derived EVs [19,28], milk EVs were found to be particularly enriched with Rabs and annexins, proteins associated with vesicle trafficking and fusion. Other than the membrane proteins with broad immunologic implications such as tetraspanins (CD9, CD63, CD81), proteins in milk EVs could be mapped to plethora of cellular pathways such as chemokine signalling, TLR signaling and T and B cell receptor signaling pathways. Furthermore, membrane proteins such as cell adhesion molecules and antigen presenting, and processing proteins were also adorned by these EVs [108].

In one of our studies to understand bovine colostrum and mature milk proteome, milk EVs were also enriched with certain milk associated proteins such as butyrophillin, xanthine oxidase, adipophilin and lactadherin. This observation further confirmed the presence of proteins implicated in eliciting immunologic reactions. Particularly, the EVs were observed to be enriched with proteins involved in platelet/neutrophil degranulation, antimicrobial peptides and complement activation [66]. Many other independent studies have focused on understanding milk EV proteome from different species such as horse, bovine and swine. In one such study, porcine milk EVs were found to contain proteins such as epidermal growth factor, thrombospondin-1, connective tissue growth factor, platelet-derived growth factor, myostatin and insulin-like growth factor-binding protein 7 among many others with roles in controlling cell proliferation [106]. Presence of milk proteins such as TGF-β [81,126], casein, lactoglobulin and lactoferrin with potential immunoregulatory roles has also been observed [107,127,128]. This further strengthens the claim that milk EVs may have a potential role in modulating the recipient’s immune system directing growth, repair and development in infants [66]. Another study to define protein cargo of human breast milk EVs concluded that they were enriched with MHCs, tetraspanins, MUC-1 and HSPs. Results from the proteomic analysis suggested a unique combination of proteins in human milk EVs; transmembrane (CD9, CD63, CD81, guanine-nucleotide binding protein), intracellular (endoplasmin, calnexin) as well as cytoplasmic (Ras-related, Rabs, annexins) [42]. Overall, milk EV protein cargo is highly diverse and differs not just between different species but also between individuals from same species. Table 1 enlists a summary of studies so far that have highlighted milk EV proteome from various species along with the major proteins implicated in the cargo. These studies suggest the ability of milk EV proteins to influence the recipient’s immunity, translation and metabolism related signaling in addition to contributing to the infant’s development and growth [106,107,108,123,129].

Milk EV transcriptome is the most widely studied cargo [111]. Several studies to date have revealed the presence of mRNA and miRNA in milk EVs with potential roles in immune modulation, growth, development and maturation of infant immune system (Table 2) [79,102,111,131]. Since, most of the miRNA found in bovine milk have either similar or even identical sequences to their human counterparts, milk consumption has been associated with the consumer’s epigenetic regulation. For instance, some of the most abundant miRNA found in bovine and human milk EVs (miR-148a, miR-21, miR-29b-1 and miR-29b-2) have a known DNMT inhibitory function [83,131]. Hence, these studies have highlighted a novel role of milk EV miRNA in gene regulation in eukaryotes both in inter-individual and cross-species. Bovine milk EVs have also been found to be highly enriched with milk protein- and ribosomal protein-related mRNAs as observed in several studies. Even though most of these mRNA were truncated, there is evidence of mRNA with a start codon to be present in bovine milk EVs, suggesting potential translation and ability to elicit immune reactions in recipient [96,102,111]. Non-coding RNA other than miRNA have also been observed to be present in bovine and human milk EVs [27,111]. The role of many of these cargo constituents of milk EVs has been discussed in more detail in further sections, in mediating inter-individual and cross-species communication.

## 3. Milk EVs in Cross-Organism and Cross-Species Communication

**Figure 4:**
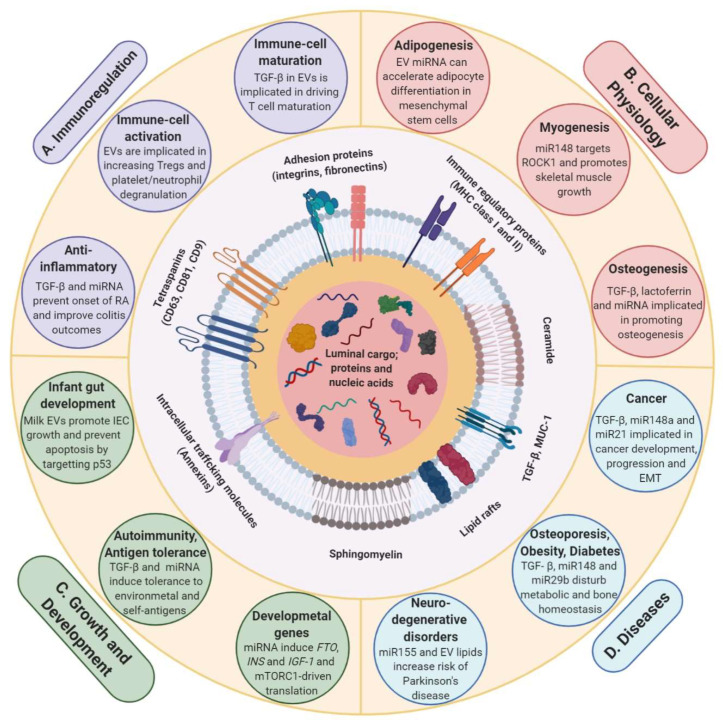
Milk EV cargo guides pathophysiological processes. The cargo of proteins, nucleic acids and lipids sequestered in milk EVs is known to mediate phenotypic changes in the recipient cells. The complexity of the cargo and the EV heterogeneity enables milk EVs to mediate an array of processes relating to; (**A**) immunoregulation, (**B**) cellular physiology, (**C**) growth and development and (**D**) diseases.

The potential role of dietary EVs in milk and their cargo in cross-organism/species communication has only been realized over the last decade. During the last few years, their importance in infant nutrition and role in physiological conditions has been studied and is becoming an increasingly important avenue of biomedical research [42,73,111]. Milk EV and its cargo is speculated to contribute to development, growth, immunity, EMT and many more pathophysiological processes [126,134,135,136] (Figure 4). For instance, infants are exposed to a vast array of foreign elements post-birth. Resistance to infections and development of a competent immune system relies heavily on factors supplied by mother’s breast milk. This intricate system comprised of cargo from metabolic, epigenetic, probiotic and stem-cell-derived system is indispensable in preventing conditions such as atopy and autoimmune diseases in later stages of life by conditioning tolerance to various antigens [90,127,137]. Furthermore, several studies have demonstrated the deficiency of bioactive miRNA and milk-derived EVs in infant formula milk [68,90]. In fact, the abundance of miRNA has been found to be highest in raw milk, followed by pasteurized milk. Whereas, formula milk has been found to be severely depleted in miRNA and proteins such as TGF-β [96,138]. Further to these findings, it has been speculated that the lack of milk-derived EVs and the associated cargo in formula milk may lead to impaired metabolic and immunologic programming in infants [127,137].

### 3.1. Milk EVs Mediate Post-Natal Development and Growth

Milk-derived EV cargo, particularly the miRNAs, are known to epigenetically regulate expression of numerous development-associated genes and signaling in infants (Figure 4).

It has been proposed that milk EV miRNA regulate expression of the three key developmental genes *FTO*, *INS* and *IGF1* and promote activation of AKT-mTORC1 pathway, leading to increased protein translation allowing for post-natal growth and species-specific metabolic programming [79,83,90]. The activation of these growth promoters is shown to be regulated via CpG demethylation, which may be mediated by the abundant miRNA in milk EVs (miR-148a, miR-152, miR-21 and miR-29s). It has been proposed that these miRNAs may play a role in the activation of specific genes by promoting demethylation at CpG islands leading to an increased expression [139,140]. For instance, regulatory role of FTO-driven transcription in post-natal growth and development has been studied in humans and mice models. Where loss-of-function mutation caused growth retardation in humans, *FTO* knockdown led to reduced weight and impaired metabolism other than retarded growth in mice models [141,142]. Similarly, promoter CpG demethylation of *INS* and *IGF1* has been shown to positively correlate with their expression [143,144]. DNMT targeting milk EV miRNA may have a role in contributing the enhanced expression of these genes, which play pivotal role in mTORC1 signaling activation and regulating postnatal growth [140]. In fact, milk consumption has been shown to cause an increase in serum levels of insulin and IGF-1 [145,146]. Overall, these observations have highlighted the importance of milk miRNA and proteins in guiding post-natal development. Based on these observations, milk EVs being abundant in these regulatory miRNAs may be speculated to guide post-natal growth and development too. However, the evidence so far is not unequivocal and needs to be supported with more studies to confirm an indispensable role of these signaling moieties. Intriguingly, nucleotide sequences coding many DNMT targeting miRNA (miR-148a-3p, miR-21–5p and miR-29b-1–3p) in bovine and human EVs share a high degree of complementarity, which also creates scope for cross-species activity of these molecules upon transfer and uptake [83].

### 3.2. Milk EVs Have Immunoregulatory Effect

Milk miRNA mediated epigenetic regulation has also been implicated to have immunomodulatory role in infants [127,137]. Immunomodulatory effects exerted by milk EVs may not be just limited to inter-organismal domain and may be observed in cross-species as well. Commercial cow milk-derived EVs that contain TGF-β have been shown to induce differentiation of naïve murine immune cells in vitro. They were able to drive the differentiation of the T cells to pathogenic Th7 lineage. Furthermore, the study also reported the uptake of bovine milk EVs by murine macrophages [81]. Whilst the protein cargo in milk EVs from humans, cows and pigs are known to be contributed majorly by mammary epithelial cells, numerous other proteins expressed and/or associated with immune cells have been discovered too [66,108,147].

Another major developmental gene that milk EV cargo is speculated to regulate through epigenetic modification is *FOXP3.* Milk EV cargo functions by inducing stable expression of *FoxP3* due to increased gene demethylation, thus enhancing the number of regulatory T cells (Tregs) [127]. Foxp3 is regarded as the primary transcription factor, driving activation of Tregs and plays a key role in inducing tolerance against not only self-antigens, critical in preventing autoimmunity, but also environmental antigens [148]. Functional analysis of human breast milk has also revealed that these vesicles repress anti-CD3-induced IL-2, IFN-γ, and TNF-α, while they increase IL-5 production from allogenic and autologous peripheral blood mononuclear cells (PBMCs). Although, it was demonstrated that milk EVs added to PBMCs were able to increase the numbers of FoxP3^+^ Tregs, the mechanism is still not completely understood [42]. However, recent studies pointed to a linear correlation between CpG demethylation of FoxP3 promoter and its expression. In this context, milk EVs containing DNMT inhibiting miRNA and TGF-β are now known to play a role in promoting stable FoxP3 expression. This might explain the role of milk in increasing Treg number and orchestrating intestinal and systemic immunity. Furthermore, milk EV TGF-β in addition to exhibiting epigenetic regulation may also exert transcriptional control by activating transcription factors SMAD2 and SMAD3, which enhance FoxP3 expression [127]. Large abundance of immune-cell related miRNA such as miR-223 has been observed in EVs derived from human milk with a potential role in immune modulation and inflammation [125,149]. In fact, four of the top ten miRNA (miR-148–3p, miR-30b-5p, miR-182–5p and miR-200a-3p) in human breast milk samples were found to be immune-based pre-miRNA [110,150].

Many other studies have indicated presence of immune regulatory cargo in milk EVs. Bovine colostrum EVs are known to contain proteins that regulate blood coagulation and platelet activation [66]. Similarly, human milk EVs are known to be an abundant source of long non-coding RNA with immune regulatory function, essential for child’s development [151]. Although, these findings have elucidated the clear role of milk EVs in immune regulation and modulation, however, their clinical utility is yet to be investigated.

### 3.3. Milk EVs Have Role in Physiological Processes, Health and Disease

The role of milk EVs is not limited to immune regulation and development. It extends beyond to various other physiological processes in infants as well as throughout adulthood. An elaborate role of milk-derived EVs has been speculated in processes such as adipogenesis [152], myogenesis [153] and osteogenesis [128] (Figure 4). For instance, milk EV cargo may be involved promoting enhanced adipogenesis via epigenetic regulation. Hypomethylation of adipogenic genes such as leptin (*LEP)* and PPAR-γ2 (*PPARG2)* is observed in uncultured adipogenic stem cells. This indicates that expression of these key adipogenic genes may be under epigenetic control [154]. In accordance with this observation, promoter hypomethylation due to inhibition of DNMT1 by miR-21, another abundant milk EV cargo, promoted adipocyte differentiation in porcine mesenchymal stem cells leading to enhanced adipogenesis [152]. DNMT1 inhibition has also been shown to promote myogenesis, another role that may be accomplished by DNMT targeting milk EV cargo. For instance, miR-148a not only promotes adipogenesis but also leads to myogenic differentiation, possibly by targeting ROCK1, which is an inhibitor of myogenesis [153]. The potential of whey protein-derived EVs in exerting increased muscle protein synthesis and anabolic effect on C2C12 myotubes has been demonstrated in vitro [134]. In line with this, another recent study has determined the effects in vivo in rats by administering bovine milk EVs and observing enhanced skeletal muscle growth [155]. Milk EV miRNA, miR-148a, miR-21 and miR-29b have been implicated recently to be also functioning by promoting osteogenic differentiation of human stem cells [83]. Corroborating this, increase in serum levels of miR-29b with increasing consumption of pasteurized bovine milk has been previously observed in humans, facilitating increased expression of osteogenic regulator Runx2 in PBMCs of the recipients [82]. More recently, the role of bovine milk EVs has been demonstrated in facilitating osteogenesis by increasing *FGF-2* and *WISP-1* expression that may also confer high Runx2 expression. Additionally, presence of Runx2 regulators such as TGF-β and lactoferrin in bovine milk EVs has also been speculated to be responsible for the increased osteoblast differentiation, but impaired bone matrix formation, in vitro [128]. The effects of bovine milk EVs in vivo were reflected via enhanced osteoclast differentiation, resulting in increased osteoclast number in female DBA1/J mice, thus confirming previous claims [156]. In conclusion, these studies have highlighted the imminent role milk EV cargo may have in driving cellular physiology. Though some of these in vitro observations have been successfully demonstrated in vivo in these studies, complete understanding of the mechanism of EVs and specific cargo exerting the effects in a living system is still lacking.

Among other functional roles of milk EVs, they have been proposed to regulate the development of infant gut by promoting IEC proliferation and survival. There is compelling evidence that bovine milk EVs are taken up by IECs, at least in vitro [114,136]. miRNA cargo from porcine milk derived EVs has been shown to enhance IEC proliferation in mice models [105]. Similarly, the potential of human breastmilk EVs in preventing IECs against oxidative stress has been demonstrated [136]. Corroborating these evidences, it has been observed that Yak milk EVs facilitate survival and proliferation of IECs in hypoxic conditions [157]. This ability of milk EVs to attenuate cell death in IECs may be because of the cargo targeting and inhibiting p53 expression [136]. Furthermore, the DNMT targeting miRNA in the milk EV cargo may have a potential role in promoting intestinal growth by enhancing lipid synthesis via upregulation of AKT and SREBP1, a key transcription factor for lipid synthesis [158].

The exemplary potential of milk EV cargo is not just limited to guiding physiological process but may also be observed in preventing infections. In this context, human milk EVs have been implicated in preventing HIV-I infection, at least in vitro. The protective role of breast milk EVs was attributed to the presence of MUC-1 on the EV surface, which enabled these nanoparticles to bind DC-SIGN on cell’s surface and compete with HIV-I particles, thus restricting infection [159,160]. However, it needs to be further studied whether such protective effects could also be observed *in vivo*. Interestingly, other protein constituents of milk such as bile-salt stimulated lipases also have been implicated to have a protective function against HIV-I infection [161]. These findings support the speculation that EVs could play a pivotal role in prevention of mother to child HIV transmission in breastfed infants.

More recently, the potential of milk EVs in influencing gut microbiota has been explored in several studies. For instance, the role of bovine milk EV cargo has been highlighted in being able to elicit changes in microflora in mice cecum [162]. Further, these vesicles could also interact with the microbiota and modulate their metabolites [163]. Another such study has revealed the ability of bovine milk EVs to restore the healthy gut bacteria and alleviate colitis induced dysbiosis [147]. Overall, these studies have highlighted the numerous health benefits which are a consequence of presence of these EVs in milk.

So far, the evidence is mounting that milk EVs have a largely beneficial role in ensuring growth and development in infants. However, continued milk consumption into adulthood may not be physiological and hence has been proposed as a reason for developing diseases, an effect thought to be mediated via milk EVs [90]. The detrimental effects of continued milk consumption may be manifested in long term and are now being better understood. Milk consumption may lead to enhanced risk of developing allergies, neurodegenerative diseases, obesity, diabetes and even cancer in later stages of life [83,90] (Figure 4). These detrimental effects have been largely attributed to miRNA and persistently high mTORC1 signaling due to continued milk consumption during adolescence [79,139]. For instance, miR-148, miR-155 and EV membrane lipids have been implicated in Parkinson’s disease pathogenesis [90]. Even though miR-148a and miR-21 have been shown to have a pivotal role in promoting adipogenesis, persistent consumption of the two miRNAs can lead to obesity. This may occur either due to adipogenic gene expression enhancement or due to imbalance in cholesterol homeostasis and paving way for metabolic imbalance [83]. Further, miR-148a and miR-21 are also known to promote expression of *FABP4*, an obesity-associated gene, by promoter hypomethylation, leading to metabolic aberrations and eventually obesity [139,140,164]. Enhanced miR-148a is also known to increase risk of osteoporosis and fractures. This may be attributed to the miRNA promoting increased adipogenesis over osteogenesis in bones, thus disturbing the bone homeostasis and increasing bone porosity [165]. miR-29 family members have also been found to be abundantly expressed in diabetes and increased levels of miR-29 in PBMCs upon consumption of cow milk can be one of the factors contributing to diabetes [82]. Lastly, miR-148a and miR-21 are also understood to be carcinogenic and are designated as ‘oncomiRs’ [83]. They are known to target tumor suppressor genes that play a role in controlling cellular processes such as proliferation, invasion and apoptosis [166,167,168,169]. Hence, it has been proposed that continued consumption of cow milk may lead to conducive tumorigenic environment resulting from activation of oncogenic signals due to increased uptake of these oncomiRs, which are abundant in milk EVs. Potential role of these miRNA in carcinogenesis has been studied in several models including colon, prostate, hepatocellular carcinoma among many others [83,90]. In addition to miRNA, high amounts of TGF-β2 in human breast milk EVs has also been shown to promote EMT in breast epithelial cells, thus increasing risk of breast cancer development and progression [126]. Milk EVs have been observed to inhibit tumor suppressor genes in normal epithelial colon cells and thus contribute to enhanced proliferation and EMT related changes. However, they failed to induce similar changes in colorectal cancer cells. Although this primarily suggests the ability of milk EVs to contribute to intestinal growth, development and repair, the possible detrimental effects due to promotion of proliferation and EMT upon continued milk consumption cannot be overlooked [117]. Corroborating this, continued consumption of milk EVs have been observed to reduce the primary tumor burden of breast cancer but accelerate metastasis (Unpublished observations, Mathivanan lab). These observations highlight the context dependent activity of milk EVs in cancer progression and indicate their ability to act as potential carcinogens via transfer of oncogenic cargo components.

## 4. Milk EVs in Therapy

The concept of utilizing EVs in therapy and treatment regimens is still nascent. Regardless, recent progress has stemmed entirely new avenues for further applications and develop EVs as drug-delivery vehicles for clinical applications. For instance, the potential of human cell- derived EVs as delivery vehicles for transporting siRNA to target the otherwise challenging target oncogenic KRAS, a key driver of pancreatic cancer, has been successfully demonstrated [170]. Many studies are now focused on understanding the physiological role of milk EVs not just in mediating cellular communication but from a therapeutic perspective. In one such study, milk EVs have been shown to be able to transport surface antibody molecules in the neonate intestine by binding the Fc receptor [171].

### 4.1. Milk EVs as Drug-Delivery Systems

Whilst therapies based on biomolecules such as RNA, recombinant proteins or drugs have shown promise, their susceptibility to degradation promoted the need for drug-delivery vehicles. Other factors such as inability to cross cell membranes, barriers and eliciting unwarranted immunological responses also limit the use of these potential therapeutics in naked form [172,173].

**Figure 5:**
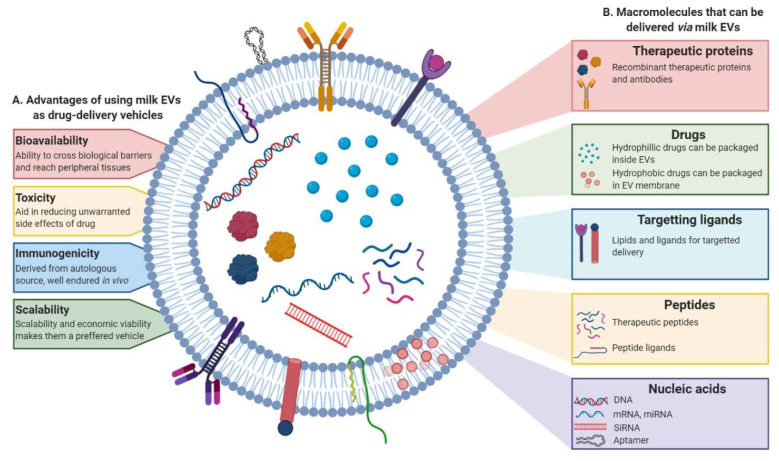
Milk EVs can be engineered to be used as drug-delivery system. Schematic representation of the (**A**) advantages of using milk EVs as drug-delivery system and (**B**) macromolecules that can be packaged and delivered via milk EVs. The nanosized vesicles, other than their intrinsic cargo, can also serve to deliver a variety of molecules including chemotherapeutic and anti-inflammatory drugs, recombinant proteins, peptides and nucleic acids. These EVs can be engineered to target specific sites of interest with the help of targeting ligands and lipids on their surface.

Although, over the last three decades intense research into nanoparticle-delivery system has led to the development of drug delivery approaches for therapy, inherent limitations have led to only a few of them making to clinics [174,175,176]. The development of a suitable nanoparticle-based delivery method, which has a decent circulating half-life, ability to evade host immune system and possess target specificity to deliver the cargo at the target site with minimal side-effects remains obscure. In this context, EVs have been demonstrated to possess numerous advantages to be exploited for the purpose of drug-delivery. Not only do they have the intrinsic ability to cross biological barriers, but also the immunogenicity and toxicity, which is a challenge with the use of synthetic drug carriers, are evaded as the EVs are sourced either from autologous or benign biological sources making them well endured in body and biofluids [171,173]. The benefit of using EVs instead of liposomes has been demonstrated in the ability of EVs to be retained longer in circulation. The presence of CD47 on EV surface renders ability to escape phagocytosis by monocytes and macrophages [170]. Furthermore, milk EVs too have been shown to be retained in the system for prolonged periods, releasing the therapeutic slowly and thus increasing efficacy, promoting enhanced cellular uptake and reducing toxic effects [177]. Biocompatibility of milk-derived EVs from various sources has been successfully demonstrated in animal models with no apparent toxicity or inflammatory responses suggesting they are well tolerated and non-immunogenic [103,119,150,177,178]. In agreement, milk EVs have been successfully deployed in pre-clinical settings as drug delivery systems (Figure 5). One such study investigated the utility of paclitaxel packaged milk EVs for oral delivery and demonstrated improved efficacy and reduced toxicity in mice xenografts [179]. Celastrol, a plant-derived triterpenoid has been shown to have therapeutic value in cancer models. However, the use of this drug is limited owing to its bioavailability and toxicity issues. Delivering celastrol loaded in bovine milk EVs has been useful in not just increasing the efficacy but reducing off-site toxicity [180]. Similarly, enhanced stability, anti-tumor activity and uptake of anti-inflammatory compound curcumin packaged in cow and buffalo milk EVs has been demonstrated [177,181]. Recent reports demonstrate an increase in the efficacy, anti-proliferative activity and reduced IC_50_ for milk EV encapsulated paclitaxel, aglycons and berry anthocyanidins in several cancer models [182,183].

In addition to the benefits mentioned before, milk EVs are a potential source to be exploited as natural drug-delivery systems given their scalability and economic viability of use [177]. Some EVs have been shown to display tissue-tropism and selectively target cells, a characteristic that can be exploited to specifically target cell types [184]. Many studies have also concluded that malignant cells, due to their leakiness, plausibly exhibit higher uptake of EVs when compared to normal cells adding to the intrinsic advantages offered by the EV-based drug delivery method [177].

### 4.2. Milk EVs as Anti-Inflammatory Agents

As milk EVs have been shown to inherently function as carriers and delivery vehicles for miRNA, siRNA and protein cargo, they may serve as an unprecedented tool for therapeutic delivery of these biomolecules in treatment of various diseases. Other than the inherent therapeutic ability of milk EVs in preventing HIV infection of immune cells, they also possess anti-inflammatory properties.

Milk EVs have been reported to mediate immunosuppressive effects in various disease models such as rheumatoid arthritis (RA) [185]. Bovine milk-derived EVs have been demonstrated to prevent onset of RA, control cartilage pathology and bone marrow inflammation in IL-1Ra-defficient mice and collagen-induced arthritis in DBA/J mice [186]. The immunosuppressive effect of bovine milk EVs has been speculated to be due to the presence of TGF-β, which may lead to diminished production of anti-collagen IgG2a accompanied by reduced splenic Th1 and Th17 mRNA [81]. Similarly, the ability of bovine milk EVs and their immune modulating proteins and miRNA cargo has also been shown in inducing anti-inflammatory effects and improving colitis outcomes [147]. It has been shown that consumption of raw cow’s milk in childhood is associated to increased FOXP3^+^ Tregs, which may lower the risk of developing atopy and asthma [187]. In this context, the role of milk EV cargo in stabilizing FOXP3 expression and promoting Treg maturation has been demonstrated, as discussed before [42]. This may enable milk EVs to induce local and systemic immunity by inducing tolerance to self as well as environmental antigens. Thus, accounting for prevention from developing atopy as well as autoimmune diseases in later stages of life [127,137].

## 5. Future Perspective

Extensive studies designed to probe the role of milk EVs in the physiological functioning and their extended utility in applications such as drug delivery and therapy has yielded many promising outcomes. These studies demonstrated that milk EVs function to protect the otherwise labile cargo of bioactive molecules sequestered within. These intrinsic properties of milk-derived EVs enable them to survive harsh, degrading conditions enabling biodistribution of the cargo to peripheral tissue. The potential of this diverse cargo carried within these signaling moieties in manifesting epigenetic regulation in the recipient is promising and has attracted interest in the field. Although preliminary evidence is mounting that milk EVs and their cargo are absorbed and elicit phenotypes in humans and animals, further proof is warranted to confirm the biological significance unequivocally. The mechanism of EV uptake and entry into systemic circulation in vivo remains obscure and is yet to be understood. In order to demonstrate in vivo bioavailability and bioactivity, it is crucial to understand the role of pathways such as transendocytosis and paracellular translocation in facilitating the milk EV uptake in gut. It is noteworthy that even though these vesicles are present in surplus in milk, physiological relevance is yet to be determined. Although the interest in understanding inter-organismal communication mediated by the milk EVs has prompted many independent studies, yet, there remains a gap of knowledge in understanding their source of origin, biogenesis and difference when compared to other vesicles and MFGs. Even though these nano-signalosomes display exemplary potential in formulating novel approaches of drug delivery and therapy further research and standardization of milk-derived EVs isolation, purity and clinical application is required for commercial implementation for therapy. Particularly, it becomes imperative to consider the deleterious consequences of milk EV cargo in eliciting diseases such as diabetes and cancer or even promoting disease progression in some cases. Several studies have suggested a context-dependent role of milk EVs in the recipient’s system. Thus, before exploiting them for therapy, it is important to address this concern about the safety of their use. Nevertheless, on the basis of what has been already uncovered, the potential of these vesicles cannot be undermined. With further investigation, it may be possible to address the current concerns regarding the bioactivity of these dietary EVs, better understand their role in physiology and pathology, and even exploit them for clinical applications.

## Figures and Tables

**Table 1:** Summary of proteomics studies for milk-derived EVs from various species.

Milk Source	Main Proteins Implicated	Method of Isolation and Characterization	Reference
Bovine	Butyrophillin, Xanthine Oxidase, Adipophilin, Lactadherin	Differential centrifugation, ultracentrifugation, sucrose gradient, LC-MS/MS	[108]
Bovine (*S. aureus* infected cows)	Butyrophillin, Xanthine dehydrogenase, Lactadherin, fatty acid synthase	Differential centrifugation, ultracentrifugation, sucrose gradient, LC-MS/MS	[130]
Human	CD9, Annexin A5, Flotillin-1, CD83, CD81, Lactadherin, Syntenin, Rab, Ras-related proteins	Differential centrifugation, ultracentrifugation, sucrose gradient, LC-MS/MS	[123]
Horse	CD81, CD63 receptors, Beta-Lactoglobulin, Lactadherin, Butyrophillin, Lactoferrin, Xanthine dehydrogenase	Differential centrifugation, ultracentrifugation, sucrose gradient, MALDI MS/MS	[107]
Porcine	CD9, CD63, HSPs, Lactadherin, Butyrophillin, Adipophilin, Xanthine oxidase	Differential centrifugation, ultracentrifugation, sucrose gradient, LC-ESI-MS/MS	[106]
Human	MHC II, CD81, MUC-1, HSPs, CD63, Butyrophillin, Lactadherin	Differential centrifugation, ultracentrifugation, sucrose gradient, LC-MS/MS	[42]
Bovine	Butyrophillin, Xanthine Oxidase, Adipophilin, Lactadherin, Rab GTPases, integrins	Differential centrifugation, ultracentrifugation, sucrose gradient, LC-MS/MS	[66]

**Table 2:** Summary of transcriptomics studies of milk-derived EVs from various species.

Milk Source	Biomolecule (Number)	Implication	Method of Isolation	Reference
Human	miRNA (602)	Immunoregulatory, infant gut development	Differential centrifugation, ExoQuick exosome precipitation	[110]
Bovine	miRNA (27)	Immune modulation	Differential centrifugation, ultracentrifugation, sucrose gradient	[132]
Bovine	mRNA (19,320), miRNA (79)	Immune modulation	Differential centrifugation, ultracentrifugation	[102]
Bovine (*S. aureus* infected cows)	miRNA (417)	Immunoregulation	Differential centrifugation, ultracentrifugation, sucrose gradient	[27]
Porcine	mRNA (19,230)	Metabolism, signalling pathways	Differential centrifugation, ultracentrifugation, sucrose gradient	[106]
Human	miRNA (330, 308)	Early infant development	Differential centrifugation, ExoQuick-TC	[118]
Bovine	miRNA (69)	Signalling pathways	Differential centrifugation, ultrafiltration, ExoEasy Maxi Kit	[133]
Bovine	miRNA (334)	Gene expression regulation	Differential centrifugation, ultracentrifugation, ultrafiltration	[125]

## Abbreviations

AKT	Protein kinase B
CD	Cluster of differentiation
DC-SIGN	Dendritic cell-specific intercellular adhesion molecule-grabbing non-integrin
DNMT	DNA methyltransferase
EGFR	Epidermal growth factor receptor
EMT	Epithelial-mesenchymal transition
EVs	Extracellular vesicles
FABP4	Fatty acid binding protein 4
FGF-2	Fibroblast growth factor 2
FoxP3	Forkhead box P3
FTO	Fat-mass and obesity associated protein
GM3	monosialodihexosylganglioside
HSP	Heat-shock protein
IECs	Intestinal epithelial cells
IFN-γ	Interferon gamma
IGF-1	Insulin-like growth factor-1
IL	Interleukin
INS	Insulin
MFG	Milk fat globule
MHC	Major histocompatibility complex
miR/miRNA	Micro RNA
mTORC1	Mammalian target of rapamycin complex 1
MUC-1	Mucin-1
MVB	Multivesicular body
PBMC	Peripheral blood mononuclear cell
ROCK1	Rho-associated, coiled-coiled containing protein kinase-1
Runx2	Runt related transcription factor 2
SMAD2/3	Mothers against decapentaplegic homolog 2/3
SREBP1	Sterol regulatory element-binding protein 1
TGF-β	Transforming growth factor-β
TLR	Toll-like receptor
TNF-α	Tumor necrosis factor-α
Tregs	Regulatory T cells
TSG101	Tumor susceptibility gene 101
WISP-1	Wnt1-inducible-signaling pathway protein-1
